# Authors’ response: Prevention of bacterial sexually transmitted infections (STI) in France: a comprehensive approach

**DOI:** 10.2807/1560-7917.ES.2019.24.12.1900187

**Published:** 2019-03-21

**Authors:** Ndeindo Ndeikoundam Ngangro, Annie Velter, Nathalie Lydie, Florence Lot

**Affiliations:** 1 Santé Publique France, Saint-Maurice, France

**Keywords:** France, STI, MSM, condoms, men who have sex with men, prevention, sexually transmitted infections

**To the editor:** We read with great interest Caumes’ letter to the editor and we are grateful for this opportunity to further explain the prevention of bacterial sexually transmitted infections (STI) in France. The author underlines rightfully that there is a missing comprehensive section dealing with STI prevention in our article titled ‘Bacterial sexually transmitted infections (STI) in France: recent trends and patients’ characteristics in 2016’ [1]. We agree that the conclusion might deal more explicitly with condom use and related topics (e.g. extensively drug-resistant (XDR) *Mycoplasma,* XDR *Neisseria gonorrhoeae*, oral-faecal transmitted diseases in men that have sex with men (MSM), recreational drug use, emergence of new STI). Nevertheless, this article does not recommend testing and treating instead of prevention since STI screening is one of the key strategies in STI prevention [1].

From an historical perspective, the dynamic of epidemics over the last two decades demonstrate that prevention coverage did not enable control of STI, despite the effectiveness of available prevention tools (e.g. condom use) [1]. Indeed, increases in STI are more noticeable in MSM, which may reflect increasing unprotected sex in this population [1-3]. An upsurge in STI is also observed in heterosexuals, with Chlamydia predominantly spreading in young women and men [1]. Thus, there are several STI epidemics driven by different determinants in various populations.

There is no controversy about condom effectiveness when it is consistently and correctly used, even if it might fluctuate by pathogens (e.g. human papillomavirus (HPV)) and sexual practices (e.g. foreplay) [4,5]. However, condom use is still inconsistent despite decades of sensitisation (e.g. awareness, barriers and enablers such as perceptions about condom use, partner support, self-efficacy, perceived risk for STI, condom costs, comfort in obtaining condoms, social norms); ongoing STI surveillance confirms that there is an insufficient level of condom use during penetrations in STI patients, regardless their sexual orientation. In addition, condoms are rarely used during oral sex in heterosexuals and MSM [6]. Factors resulting in less condom use therefore need to be carefully addressed through behavioural interventions and alternative prevention tools [7].

The role of asymptomatic and extra genital STI in sustaining silent epidemics should also be taken into account. Nucleic acid amplification test (NAAT) and testing recommendations have improved detection of asymptomatic and symptomatic STI and contribute to increasing number of diagnoses [1,8]. However, the volume of diagnosed and treated STI likely remains insufficient to control ongoing epidemics, even combined with present level of condom use [1].

Consequently, prevention should target the main drivers of STI epidemics to reduce STI reservoirs as well as new acquisitions. Condoms remain a corner stone in STI prevention and Santé publique France therefore actively promote their use through social marketing campaigns, free provision of condoms, sexual health education and promotion and collaborations with stakeholders (e.g. clinicians, scientists and patients' associations).

Considering the burden of STI, invisible epidemics of undiagnosed STI, threat of multidrug resistance and the ‘reoccurrence of severe complications’ and ‘high rate of sequelae’ mentioned by Caumes, a comprehensive approach including several levels of prevention is required to control STI [8-11]. This justifies a wide range of combined interventions such as condom reimbursement by the national health insurance, systematic Chlamydia testing in young women and men, STI clinics targeting the most exposed or vulnerable populations, test reimbursements and sexual partners notification in the framework of a national sexual health strategy [1,11-13].

Concerning HIV pre-exposure prophylaxis (PrEP) patients’ follow-up primarily targets HIV transmission, but its components including sexual health counselling and STI testing can contribute to diagnosing invisible STI in highly exposed MSM. This could lead to a decrease in STI on the long-term if ‘more patients are treated and transmission chains are broken’ after an expected initial rise in the number of diagnoses [14,15]. We also must remember the role of STI in HIV acquisition and how valuable PrEP consultations are in preventing HIV transmission in highly exposed populations.

In conclusion, condom use remains a key component of STI prevention. Nevertheless, a comprehensive approach of STI prevention should continue, including testing and treating patients, notification of sexual partners following diagnosis, updating national guidelines and antimicrobial surveillance to prevent occurrence of multidrug-resistant pathogens, reinforce surveillance to detect emergent STI, understanding risk behaviour patterns, identifying barriers and finding means to improve access to a wide range of prevention tools.

## References

[1] Ndeikoundam Ngangro N Viriot D Fournet N Pioche C De Barbeyrac B Goubard A Bacterial sexually transmitted infections in France: recent trends and patients’ characteristics in 2016. Euro Surveill. 2019;24(5):1800038. 10.2807/1560-7917.ES.2019.24.5.1800038 30722812 PMC6386212

[2] Velter A Saboni L Sommen C Bernillon P Bajos N Semaille C Sexual and prevention practices in men who have sex with men in the era of combination HIV prevention: results from the Presse Gays et Lesbiennes survey, France, 2011. Euro Surveill. 2015;20(14):21090. 10.2807/1560-7917.ES2015.20.14.21090 25884150

[3] Méthy N Meyer L Bajos N Velter A Generational analysis of trends in unprotected sex in France among men who have sex with men: The major role of context-driven evolving patterns. PLoS One. 2017;12(2):e0171493. 10.1371/journal.pone.0171493 28170424 PMC5295686

[4] Holmes KK Levine R Weaver M Effectiveness of condoms in preventing sexually transmitted infections. Bull World Health Organ. 2004;82(6):454-61. 15356939 PMC2622864

[5] WellerSDavisK Condom effectiveness in reducing heterosexual HIV transmission. Cochrane Database Syst Rev. 2002;1(1):CD003255. 10.1002/14651858.CD00325511869658

[6] Santé publique France. Bulletin des réseaux de surveillance des IST : données 2014. [Bulletin of STI surveillance networks: 2014]. Saint Maurice : Santé publique France; 2015. French. Available from: http://invs.santepubliquefrance.fr/fr,/Dossiers-thematiques/Maladies-infectieuses/VIH-sida-IST/Infections-sexuellement-transmissibles/Bulletins-des-reseaux-de-surveillance-des-IST/Donnees-au-31-decembre-2014

[7] Kersaudy-Rahib D Lydié N Leroy C March L Bébéar C Arwidson P Chlamyweb Study II: a randomised controlled trial (RCT) of an online offer of home-based *Chlamydia trachomatis* sampling in France. Sex Transm Infect. 2017;93(3):188-95. 10.1136/sextrans-2015-052510 28377422

[8] Caumes E Réémergence inquiétante des infections sexuellement transmissibles dans un contexte de promotion de la PrEP. Pourquoi ne pas en revenir au safer sex? [Alarming reemergence of sexually transmitted infections in the PrEP area: Why not come back to safer sex?]. Presse Med. 2018;47(9):719-21. 10.1016/j.lpm.2018.09.007 30368404

[9] Ndeikoundam Ngangro N, Bouvet De La Maisonneuve P, Le Strat Y, Fouquet A, Viriot D, Fournet N, et al. Estimations nationales et régionales du nombre de diagnostics d’infections à Chlamydia et à gonocoque en France en 2016. [Estimates of the national and sub-national number of diagnoses of chlamydial and gonococcal infections in France in 2016]. Saint Maurice: Santé publique France; 2018. French. Available from:http://invs.santepubliquefrance.fr/fr,/Publications-et-outils/Rapports-et-syntheses/Maladies-infectieuses/2018/Estimations-nationales-et-regionales-du-nombre-de-diagnostics-d-infections-a-Chlamydia-et-a-gonocoque-en-France-en-2016

[10] Poncin T Fouere S Braille A Camelena F Agsous M Bebear C Multidrug-resistant *Neisseria gonorrhoeae* failing treatment with ceftriaxone and doxycycline in France, November 2017. Euro Surveill. 2018;23(21):1800264. 10.2807/1560-7917.ES.2018.23.21.1800264 29845928 PMC6152217

[11] Haute Autorité de Santé. Réévaluation de la stratégie de dépistage des infections à Chlamydia trachomatis. [Evaluation of the screening strategy for Chlamydia trachomatis infection]. Saint Denis: Haute Autorité de Santé; 2007. French. Available from: https://www.has-sante.fr/portail/upload/docs/application/pdf/2018-10/recommandation_en_sante_publique__reevaluation_de_la_strategie_de_depistage_des_infection_a_chlamydia_trachomatis_vf.pdf

[12] Ministère des solidarités et de la santé. The National Sexual Health Strategy, 2017-2030 agenda. Paris: Ministère des solidarités et de la santé; 2017. Available from: https://solidarites-sante.gouv.fr/IMG/pdf/strategie_sante_sexuelle_def_ang.pdf

[13] Ministère des solidarités et de la santé. Premier préservatif remboursé par l’Assurance maladie.[First condom reimbursed by health insurance]. Paris: Ministère des solidarités et de la santé; 2018. French. Available from: https://solidarites-sante.gouv.fr/actualites/presse/communiques-de-presse/article/premier-preservatif-rembourse-par-l-assurance-maladie

[14] Jenness SM Weiss KM Goodreau SM Gift T Chesson H Hoover KW Incidence of Gonorrhea and Chlamydia Following HIV Preexposure Prophylaxis among Men Who Have Sex with Men: A Modeling Study. Clin Infect Dis. 2017;65(5):712-8. 10.1093/cid/cix439 28505240 PMC5848234

[15] Werner RN Gaskins M Nast A Dressler C Incidence of sexually transmitted infections in men who have sex with men and who are at substantial risk of HIV infection - A meta-analysis of data from trials and observational studies of HIV pre-exposure prophylaxis. PLoS One. 2018;13(12):e0208107. 10.1371/journal.pone.0208107 30507962 PMC6277101

